# Impacts of Kinematic Information on Action Anticipation and the Related Neurophysiological Associations in Volleyball Experts

**DOI:** 10.3390/brainsci14070647

**Published:** 2024-06-27

**Authors:** Xizhe Li, Danlei Wang, Siyu Gao, Chenglin Zhou

**Affiliations:** School of Psychology, Shanghai University of Sport, Shanghai 200438, China; lxz18116150115@163.com (X.L.); sus_wdl@163.com (D.W.); gaosiyu_carol@163.com (S.G.)

**Keywords:** volleyball spike, action anticipation, information quantity, EEG technology

## Abstract

In this study, we investigated the cognitive mechanisms underlying action anticipation in volleyball players, especially concerned with the differences between experts and amateurs. Participants included both expert (male, *N* = 26) and amateur (male, *N* = 23) volleyball players, who were asked to predict spiking movements containing high, medium, and low levels of kinematic information while their electrophysiological activities were recorded. The high-information stimuli included the whole spiking action, the medium-information stimuli ended at 120 ms, and the low-information stimuli ended at 160 ms before hand–ball contact. The results showed that experts significantly outperformed amateurs in both prediction accuracy (68% in experts vs. 55% in amateurs) and reaction time (475.09 ms in experts vs. 725.81 ms in amateurs) under the medium-information condition. Analysis of alpha rhythm activity revealed that experts exhibited the strongest desynchronization under the low-information condition, suggesting increased attentional engagement. In contrast, amateurs showed the weakest desynchronization under the medium-information condition. Furthermore, mu rhythm activity analysis showed greater desynchronization in the duration of 100–300 ms before hand–ball contact for experts, correlating with their higher anticipation accuracy. These findings highlight the significant kinematic information-processing abilities of volleyball experts and elucidate the neural mechanisms underlying efficient attentional engagement and mirroring. Therefore, this study provides valuable insights for the development of targeted training programs through which to enhance athletic performance.

## 1. Introduction

Recent studies demonstrate that the exceptional performance of high-level athletes stems not only from their proficient motor skills but also from their cognitive development [1,2]. In highly competitive sports, the accurate prediction of opponents’ actions is crucial; experts utilize early movement cues to make predictions, while novices predominantly rely on later object cues, such as ball trajectory [3]. Rapid and precise action anticipation provides elite players with crucial time to plan and execute their responses [4]. Thus, the ability to anticipate actions is key to victory and serves as an indicator of an athlete’s proficiency [3,5].

In volleyball competitions, the rapid transitions between offense and defense, coupled with intricate strategies, require athletes to anticipate each hit within a constrained timeframe. Studies consistently underscore the remarkable action anticipation abilities of volleyball experts [6,7,8]; however, debates still remain regarding the extent of volleyball experts’ anticipatory abilities. Using the temporal occlusion paradigm, the authors of [9] found that expert players and coaches outperformed referees and novices in anticipating attack directions only during late occlusion. Conversely, other studies have found that experts also surpass novices in early occlusions, due to their proficiency in recognizing early movement patterns [6,7,10]. To elucidate this matter, it is important to distinguish between elite and intermediate players. Unlike novices without any relevant domain-specific experience, studies indicate that intermediate players can also utilize movement information for anticipation, albeit not as proficiently as experts [5,11]. Thus, comparing expert and amateur players can further explore how crucial information is utilized during action anticipation and shed light on the mechanisms underlying the anticipation abilities of elite volleyball players.

One possible factor contributing to experts’ anticipatory advantage is the effective utilization of kinematic information from movements [12]. Experts often use visual input more effectively when scanning and extracting movement information [13,14,15]. Research has shown that only volleyball athletes can make accurate predictions based on body kinematics in their sport [8], and recognizing crucial body movement patterns can automatically aid in preparatory motor activation [16]. Using gaze-tracking techniques, studies found that elite volleyball players exhibit fewer fixations and longer durations, indicating a stable visual search pattern and the ability to efficiently extract crucial task-relevant information [17,18]. Studies using electrophysiology or neuroimaging techniques suggest that this kinematic processing advantage may stem from a special “mirroring” mechanism. When volleyball players observe and predict domain-specific movements, nodes of the action observation network, such as the supplementary motor area and the superior parietal lobule, exhibit greater responses compared to controls [6]. This activation of the mirror neuron system can also be reflected in sensorimotor cerebral electroencephalography (EEG) oscillations around 8–13 Hz, known as mu rhythm suppression [19]. A study involving table tennis players found stronger mu rhythm desynchronization when athletes observed opponents’ movements and imagined their own responses [20]. Mu rhythm activities could also predict reactions in other motor tasks, such as golf putts [21].

Another potential factor contributing to experts’ anticipatory advantage is their attentional recruitment and allocation capacity. A study investigating the perceptual and cognitive skills of volleyball players found that experts exhibited superior speed in a visuo-spatial attentional processing task [17]. Additionally, when movement stimuli were presented in the foveal, peripheral, or both fields, only highly skilled volleyball players showed a benefit from stimuli from both fields, compared to either source in isolation, and demonstrated better anticipation performance than novices [7]. One neurophysiological indicator of this attentional advantage is alpha rhythm desynchronization [22], which reflects cortical information processing in elite athletes [23]. Studies have noted that experts show lower alpha desynchronization, indicating a neural efficiency advantage [24,25,26]. Notably, only highly skilled volleyball players exhibited right parietal lateralization of alpha activity and greater alpha power, compared to less-skilled players, when predicting volleyball attacks [27]. 

In this study, in order to further investigate the cognitive mechanisms underlying the action anticipation advantage of elite volleyball players, we compared the performance of volleyball experts and amateurs in an action anticipation task and analyzed the relevant mu and alpha rhythm activities. Given that spiking is the most prevalent and important scoring approach in volleyball, requiring a rapid response, we used spiking movements with three different levels of kinematic information to identify experts’ primary advantage. We hypothesized that experts would outperform amateurs, particularly in predicting actions with medium information, and would exhibit distinct neural oscillation patterns in mu and alpha rhythms indicative of the effective kinematic processing and attentional engagement occurring.

## 2. Materials and Methods

### 2.1. Participants

This study included a mixed design of 2 groups (expert and amateur) × 3 levels of information volume (high, medium, and low), and used G-power software (version 3.1.9.2) to calculate the required sample size. With an effect size of 0.25, a confidence level of 0.95, a statistical power of 0.9, and a correlation coefficient for repeated measures of 0.5, a minimum sample of 44 participants was needed for a 2 × 3 repeated measures ANOVA. Statistical analysis was conducted using IBM SPSS Statistics, Version 26.0 (IBM SPSS, Inc., Chicago, IL, USA). A repeated measures analysis of variance (ANOVA) was performed to examine the accuracy and RTs, using the group (experts vs. amateurs) as a between-subjects factor and the condition (Information: low, medium, or high) as a within-subjects factor. Twenty-six high-level volleyball players (age: 20.38 ± 1.82; range 18–26 years), qualified as Grade 1 Athletes, were recruited as experts. As shown in Table 1, twenty-three Shanghai Sport University volleyball class students (age: 21.26 ± 2.33; range 18–26 years) were recruited as amateurs. All participants were right-handed, had no psychological or neurological health issues, and voluntarily took part in the experiment. Our study was approved by the local ethics committee (No. 102772023PT197) and complied with the principles of the revised Helsinki Declaration. All of the participants signed informed consent forms and received remuneration.

### 2.2. Materials

**Figure 1:**
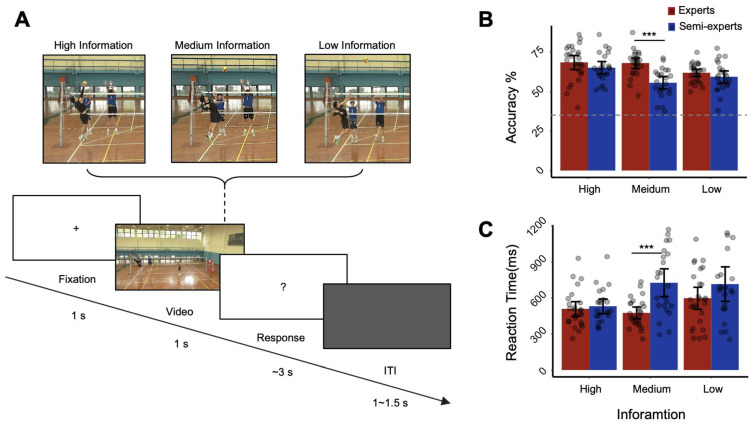
Experimental procedure and behavioral results. (**A**) Schematic diagram of experimental materials. The top shows video material fragments with three different amounts of Information: high, medium, and low; the bottom shows the flow chart of a single trial in the experiment. (**B**) Accuracy. (**C**) Reaction time. The error bar represents the 95% confidence interval. * *p* < 0.05; ** *p* < 0.01; *** *p* < 0.001.

The experimental stimuli were obtained from recordings of spiking movements in a high-level volleyball training session. Videos depicting a male volleyball player spiking, with an equal probability of serving to the left or right, were recorded from the perspective of his opponent (Canon 5D Mark III; resolution, 1280 × 720 pixels). The captured videos were processed using Adobe Premiere software (Adobe Systems Incorporated San Jose, CA, USA). A spiking movement mainly comprises three segments: jump, arm swing, and ball contact. Each spiking video was edited into three Information conditions: high, medium, and low. High-information videos ended when the spiker hit the ball, thus containing all the kinematic information present in the video. Medium-information videos ended at 120 ms before contact, containing most of the arm swing. Low-information videos ended at 160 ms before contact, containing only the initial part of the arm swing (Figure 1A). The duration of each clip was 1000 ms; in total, 80 clips were included in the experiment.

### 2.3. Design and Procedure

This study employed a 2 × 3 mixed design, with two groups (experts and amateurs) and three levels of information (high, medium, and low). The laboratory was consistently set up to ensure an enclosed, noise-free environment with soft lighting. Prior to the experiment, each participant was required to thoroughly clean and dry their scalp to remove any residue, and turn off mobile phones and other communication devices to avoid electromagnetic interference. Participants were comfortably seated at a desk, with the chair height adjusted to align their line of sight with the center of the screen. After these preparations, the experimental procedure was explained in detail and demonstrated. Throughout the experiment, participants were asked to concentrate and minimize blinking. The experimental stimuli were presented on a 19-inch LCD monitor with a refresh rate of 60 Hz and a resolution of 1920 × 1080.

During the experiment, participants were instructed to watch the videos attentively to understand how key offensive plays were handled, observing changes in the attacker’s footsteps, run-up, jump, arms, and hands. E-prime, version 3.0 (Psychology Software Tools, Inc., Sharpsburg, PA, USA), was used to present all visual stimuli with the corresponding triggers and to record both response accuracy and reaction time via a key press. Each trial started with a fixation screen for 1000 ms. Thereafter, participants were required to watch videos from three different information levels for 1000 ms. After each video ended, they had up to 3000 ms to respond by pressing one of three keys to indicate a straight (1), middle (2), or diagonal (3) attack direction. Participants were asked to use their right index finger to press 1 for straight, 2 for middle, or 3 for oblique. Each trial ended with a gray blank screen with a random duration between 1000 and 1500 ms. There was a practice session with six trials to allow participants to become familiar with the experimental procedure before the formal task, in which each participant completed 80 trials per information condition. 

### 2.4. Data Analysis

#### 2.4.1. Behavioral Data Analysis

Accuracy and reaction time were used as the indices of anticipation task performance. The reaction time was measured from the end of the video, and trials with a response time exceeding 3000 ms were excluded. 

#### 2.4.2. EEG Data Acquisition and Preprocessing

EEG data were collected using a 64-channel actiCAP system compliant with the international 10-10 system (Brain Products GmbH, Gilching, Germany) and recorded using Brain Vision Recorder 2.0 (Brain Products GmbH, Germany) at a sampling rate of 1000 Hz. The FCz electrode served as the reference, and GND as the ground electrode. Two electrodes were placed 1 cm lateral to the left eye and 1 cm below the right eye to record horizontal and vertical eye movements, respectively. No online filtering was used during recording.

EEG data analysis was conducted using the software packages EEGLAB and Fieldtrip of MatlabR2013b (MathWorks Inc., MA, USA). The EEG data processing steps included the following: (1) visual inspection of data to identify and interpolate bad channels; (2) application of a Butterworth filter with a passband of 0.1 to 30 Hz; (3) separation of EEG components using Independent Component Analysis (ICA) to manually exclude ocular artifacts; (4) epoching data, according to the fixation onset, from −1000 ms to 2000 ms; (5) automated artifact rejection to remove trials with amplitudes exceeding ±100 μV; (6) re-referencing using the average of all electrodes and restoring the FCz point; (7) averaging the EEG data of each experimental condition; (8) baseline correction using data starting from 500 ms prior to the fixation onset; and (9) employing the wavelet transform for time–frequency analysis, with the output set to power spectral density (PSD), measured in microvolts squared (µV^2^). To enhance computational efficiency, the data were zero-padded to the next power of two (‘nextpow2’). The analysis was conducted over a frequency range of 3 Hz to 40 Hz, with a step size of 0.5 Hz. The time window for analysis spanned from −1 s to 2 s, with a step size of 0.02 s. Additionally, the wavelet window lengths were set to an equally spaced sequence, ranging from 3 to 12, corresponding to the frequency range of the analysis, thereby increasing the wavelet window length with increasing frequency.

#### 2.4.3. EEG Data Time–Frequency Analysis

Mu rhythm refers to the synchronized neural activity in the sensorimotor cortex within the 8–13 Hz range, which shows a decrease in power during action observation and execution, known as desynchronization. In previous studies [28], mu rhythm analysis typically involved central region electrodes (C3, Cz, and C4), while alpha rhythm (9–12 Hz) analysis involved occipital electrodes (Oz, O1, and O2). Given our focus on the electrophysiological characteristics prior to and during contact with the ball after the jump, we selected a window of analysis from 500 ms to 1000 ms, with a 100 ms set (T1–T5), after the video onset for this study. Power values were transformed into decibels by multiplying the log ratio by a factor of 10 (Grandchamp and Delorme, 2011) and averaging cross-selected electrodes. Repeated measures ANOVAs were conducted with the between-subject factor (groups: experts, novices) and two within-subject factors (information levels: high, medium, low; time windows: T1–T5) for alpha and mu activities.

#### 2.4.4. Statistical Analysis

For the behavioral data, the reaction times and accuracy rates of all the participants were imported into SPSS for statistical analysis. A 2 (groups) × 3 (information levels) repeated measures ANOVA was conducted, for which the significance threshold was set at *p* < 0.05. For the EEG data, we first identified the time and frequency ranges of interest from the processed time–frequency information. Specifically, we focused on the frequency information between 500 ms and 1000 ms post-stimulus, dividing this interval into five 100 ms segments. The mean frequency for each participant within each segment was calculated using MATLAB. Consequently, each participant retained mean frequency data for five segments under each condition. These data were then imported into SPSS for a 2 (Group) × 3 (Information) × 5 (Time) repeated measures ANOVA, for which the significance threshold was set at *p* < 0.05.

## 3. Results

### 3.1. Behavioral Results

#### 3.1.1. Accuracy

As shown in Table 2, the anticipation accuracy results showed that group had a significant main effect (*F* _1,47_ = 9.532, *p* = 0.003, *η*^2^*p* = 0.169), with the expert group demonstrating significantly higher accuracy than the amateur group. Information quantity also had a significant main effect, (*F* _2,94_ = 11.430, *p* < 0.001, *η*^2^*p* = 0.196). Furthermore, the interaction between group and information quantity was also significant (*F*_2,94_ = 7.653, *p* = 0.001, *η*^2^*p* = 0.140).

The simple effects analysis showed that, under the medium-information condition, the accuracy of the expert group was significantly higher than that of the amateur group (*p* < 0.001, 95% CI [7.433, 17.256]). There was no significant difference in accuracy between the expert and amateur groups under either the high-information condition (*p* = 0.258, 95% CI [−9.260, 2.540]) or the low-information condition (*p* = 0.240, 95% CI [−1.807, 7.043]). The expert group showed no significant differences between high- and medium-information conditions (*p* = 1, 95% CI [−3.975, 5.028]), but significant differences were found between high- and low-information conditions (*p* = 0.009, 95% CI [1.373, 11.858]), and between medium- and low-information conditions (*p* = 0.003, 95% CI [1.759, 10.419]). The amateur group showed significant differences between high- and medium-information conditions (*p* < 0.001, 95% CI [4.725, 14.297]), and significant differences between high- and low-information conditions (*p* = 0.036, 95% CI [0.299, 11.45]), but no significant differences between medium- and low-information conditions (*p* = 0.167, 95% CI [−8.241, 0.966]).

These results suggest that medium information is a critical factor for the accuracy of action anticipation tasks, and it represents a perceptual prediction advantage for expert athletes.

#### 3.1.2. Reaction Time

The results of the volleyball spike prediction task were analyzed by conducting a 2 (Group) × 3 (Information quantity) repeated measures ANOVA on the volleyball spike action anticipation task, revealing a significant main effect from group (*F* _1,47_ = 7.114, *p* = 0.010, *η*^2^*p* = 0.131), with the expert group having significantly shorter reaction times than the amateur group. There was also a significant main effect from information quantity (*F*_2,94_ = 8.402, *p* = 0.001, *η*^2^*p* = 0.152), with the expert group showing significantly shorter reaction times under medium-information conditions compared to high- and low-information conditions, and the amateur group showing significantly shorter reaction times under high-information conditions compared to medium- and low-information conditions. The interaction between group and information quantity was also significant (*F*_2,94_ = 5.779, *p* = 0.008, *η*^2^*p* = 0.109). 

Further simple effects analysis indicated that, under medium-information conditions, the reaction times of the expert group were significantly shorter than those of the amateur group (*p* < 0.001, 95% CI [133.870, 367.555]). There were no significant differences in reaction times between the expert and amateur groups under high-information conditions (*p* = 0.613, 95% CI [−107.538, 64.102]) or low-information conditions (*p* = 0.149, 95% CI [−281.227, 44.184]). The expert group showed no significant differences in reaction times between high- and medium-information conditions (*p* = 1, 95% CI [−60.671, 124.715]), nor between high- and low-information conditions (*p* = 0.369, 95% CI [−230.421, 51.808]); however, there were significant differences between medium- and low-information conditions (*p* = 0.020, 95% CI [15.553, 227.104]). The amateur group showed significant differences between high- and medium-information conditions (*p* < 0.001, 95% CI [98.420, 295.526]), and between high- and low-information conditions (*p* = 0.010, 95% CI [36.075, 336.145]); however, there were no significant differences between medium- and low-information conditions (*p* = 1, 95% CI [−101.599, 123.326]). These results indicate that expert-level athletes exhibit the shortest reaction times in action anticipation tasks when provided with medium information, with the longest reaction times occurring under low-information conditions, thus demonstrating that the amount of information is a key factor affecting reaction times in action anticipation tasks.

### 3.2. Alpha Rhythm Study Results

**Figure 2:**
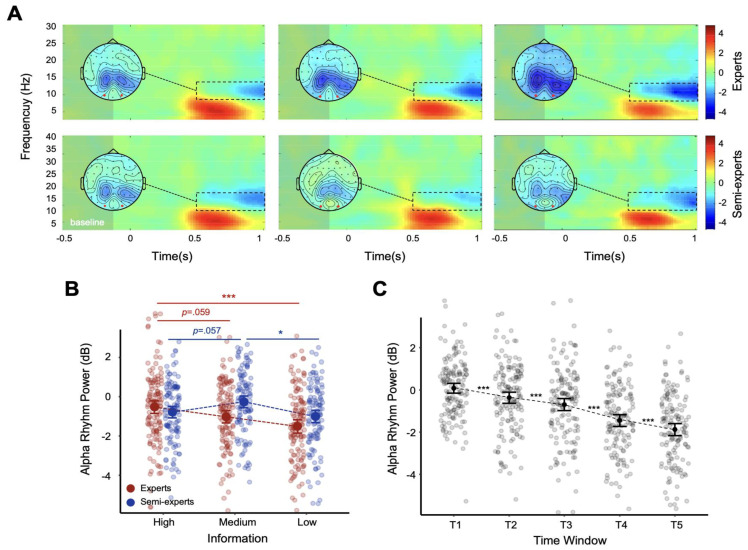
Alpha oscillations. (**A**) Time−frequency representation and topography plots of alpha oscillations under high—(**left**), medium—(**middle**), and low—(**right**) information conditions. The time–frequency windows for analysis are outlined with dotted lines. The red circles on the topographic map indicate the analyzed channels (O1, Oz, and O2). (**B**) The Group × Information interaction of the alpha oscillation and (**C**) alpha oscillations under different time windows (T1–T5). * *p* < 0.05; ** *p* < 0.01; *** *p* < 0.001.

Alpha rhythms were analyzed using a 2 (Group: expert, amateur) × 3 (Information: low, medium, high) × 5 (Time window: T1–T5) repeated measures ANOVA. (Figure 2C) The results showed that the main effect from time window was significant (F _4,188_ = 108.228, *p* < 0.001, *η*^2^*p* = 0.697), and the paired comparison of time windows showed that T1 and T2 were significant (*p* < 0.001, 95% CI [0.176, 0.704]); T1 and T3 were significant (*p* < 0.001, 95% CI [0.397, 1.131]); T1 and T4 were significant (*p* < 0.001, 95% CI [1.089, 1.945]); T1 and T5 were significant (*p* < 0.001, 95% CI [1.504, 2.375]); T2 and T3 were significant (*p* < 0.001, 95% CI [0.112, 0.535]); T2 and T4 were significant (*p* < 0.001, 95% CI [0.768, 1.386]); T2 and T5 were significant (*p* < 0.001, 95% CI [1.166, 1.832]); T3 and T4 were significant (*p* < 0.001, 95% CI [0.557, 0.949]); T3 and T5 were significant (*p* < 0.001, 95% CI [0.871, 1.481]); T4 and T5 were significant (*p* < 0.001, 95% CI [0.871, 1.481]); and T4 and T5 were significant (*p* < 0.001, 95% CI [0.220, 0.625]). 

The main effect from information volume was significant (F _2,94_ = 9.818, *p* < 0.001, *η*^2^*p* = 0.173), and the paired comparison of information volume showed that there was significance between high information volume and low information volume (*p* = 0.002, 95% CI [0.200, 1.006]); between the medium and low information volumes (*p* = 0.002, 95% CI [0.206, 1.034]); the main effect from group was not significant, (F _1,47_ = 0.719, *p* = 0.401, *η*^2^*p* = 0.015); the interactions among information volume, time window, and group were not significant (F _8,376_ = 0.555, *p* = 0.815, *η*^2^*p* = 0.012); and the interaction between information volume and group was significant, (F _2,94_ = 5.521, *p* = 0.005, *η*^2^*p* = 0.105). 

Further simple effect analysis showed that, under the conditions of high, medium, and low information volume, there was no significant difference in alpha rhythm between the expert and amateur groups (*p* = 0.557, 95% CI [−0.623, 1.143]; *p* = 0.078, 95% CI [−1.605, 0.089]; *p* = 0.262, 95% CI [−1.399, 0.390]). As shown in Figure 2B, there were significant differences between experts under high- and low-information conditions (*p* < 0.001, 95% CI [0.434, 1.537]) and amateurs under medium- and low-information conditions (*p* = 0.011, 95% CI [0.143, 1.350]).

### 3.3. Mu Rhythm Study Results

Mu rhythms were analyzed using a 2 (Group: expert, amateur) × 3 (Information: low, medium, high) × 5 (Time window: T1–T5) repeated measures ANOVA. The results showed a significant main effect of information quantity (*F*_2,94_ = 8.95, *p* < 0.001, *η*^2^*p* = 0.160). Pairwise comparisons indicated significant differences between high- and low-information conditions (*p* = 0.008, 95% CI [0.125, 1.044]) and between medium- and low-information conditions (*p* = 0.001, 95% CI [0.226, 1.064]). A significant main effect of the time window was also observed, (*F*_4,188_ = 91.402, *p* < 0.001, *η*^2^*p* = 0.660). Pairwise comparisons within time windows showed significant differences between T1 and T2 (*p* < 0.001, 95% CI [0.360, 0.880]), T1 and T3 (*p* < 0.001, 95% CI [0.638, 1.477]), T1 and T4 (*p* < 0.001, 95% CI [1.089, 1.980]), T1 and T5 (*p* < 0.001, 95% CI [1.367, 2.178]), T2 and T3 (*p* < 0.001, 95% CI [0.221, 0.654]), T2 and T4 (*p* < 0.001, 95% CI [0.588, 1.240]) T2 and T5 (*p* < 0.001, 95% CI [0.870, 1.435]), T3 and T4 (*p* < 0.001, 95% CI [0.273, 0.680]), T3 and T5 (*p* < 0.001, 95% CI [0.478, 0.952]), and T4 and T5 (*p* = 0.001, 95% CI [0.075, 0.402]). No significant main effect from group was found, (*F*_1,47_ = 2.938, *p* = 0.093, *η*^2^*p* = 0.059), and there was no significant interaction among information quantity, time window, and group (*F*_8,376_ = 1.198, *p* = 0.299, *η*^2^*p* = 0.025).

Further simple effects analyses showed a significant difference in mu rhythms between expert and amateur groups under the T3 and T4 time windows (*p* = 0.044, 95% CI [0.026, 1.789]; *p* = 0.044, 95% CI [0.024, 1.735]). Experts showed significance between the T1 and T2 (*p* < 0.001, 95% CI [0.451, 1.164]), T1 and T3 (*p* < 0.001, 95% CI [0.847, 1.995]), T1 and T4 (*p* < 0.001, 95% CI [1.273, 2.494]), T1 and T5 (*p* < 0.001, 95% CI [1.469, 2.581]), T2 and T3 (*p* < 0.001, 95% CI [0.317, 0.909]), T2 and T4 (*p* < 0.001, 95% CI [0.630, 1.522]), T2 and T5 (*p* < 0.001, 95% CI [0.831, 1.604]), T3 and T4 (*p* < 0.001, 95% CI [0.183, 0.742]), and T3 and T5 (*p* < 0.001, 95% CI [0.279, 0.929]) time windows. There were also significant differences between the T2 and T4 (*p* < 0.001, 95% CI [0.278, 1.227]), T2 and T5 (*p* < 0.001, 95% CI [0.676, 1.499]), T3 and T4 (*p* < 0.001, 95% CI [0.194, 0.788]), T3 and T5 (*p* < 0.001, 95% CI [0.480, 1.172]), and T4 and T5 (*p* = 0.001, 95% CI [0.097, 0.574]) time windows for the amateur group.

### 3.4. Correlation Analysis

**Figure 3:**
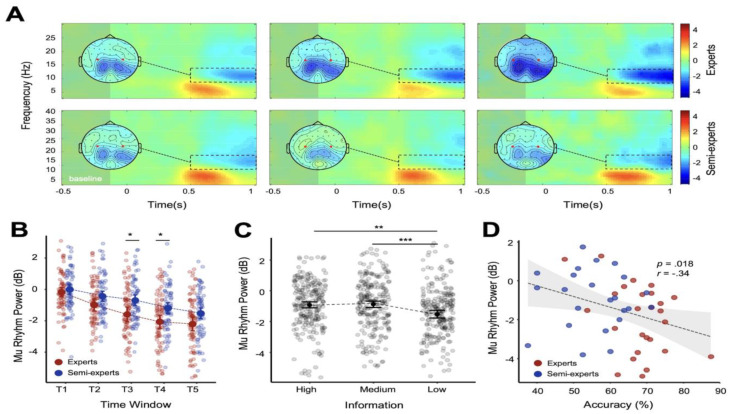
Mu oscillation results. (**A**) Time–frequency representation and topography plots of mu oscillations under the high—(**left**), medium—(**middle**), and low—(**right**) information conditions. The time–frequency windows for analysis are outlined with dotted lines. The red circles on the topographic map indicate the analyzed channels (C3, Cz, and C4). (**B**) The change in mu rhythms between experts and amateurs in different time windows, with red indicating experts and blue indicating amateurs. (**C**) Graph of pairwise comparisons of Information volume. (**D**) Correlation between T3 and T4 time window mu rhythm activity under the medium-information condition. * *p* < 0.05; ** *p* < 0.01; *** *p* < 0.001.

In order to explore the relationship between the anticipatory advantage and relevant neural activities in experts, Pearson’s correlations between task performance and EEG synchronizations under the medium-information condition were conducted. The results showed that the intensity of mu synchronization in the T3 and T4 time windows was negatively correlated with accuracy (r = −0.372, *p* = 0.009; r = −0.337, *p* = 0.018; Figure 3D). The intensity of alpha synchronization showed no significant correlation.

## 4. Discussion

In this study, we investigated the action anticipation advantage of volleyball experts using the EEG technique. The results indicated significant differences in accuracy and response time between experts and amateurs under the medium-information condition. Additionally, notable differences in mu and alpha rhythms were observed, reflecting distinct attentional engagement and mirroring activity patterns between the two groups.

The behavioral results highlight the critical role of kinematic information in action anticipation among volleyball players, supporting our hypothesis. Experts exhibited significantly higher accuracy and shorter reaction times compared to amateurs when predicting medium-information actions, suggesting that experts can maintain high performance even when kinematic information is incomplete, likely due to their extensive experience and well-developed anticipatory skills. Expert-level athletes exhibit the shortest reaction times in action anticipation tasks when provided with medium information, with the longest reaction times occurring under low-information conditions, which demonstrates that the amount of information is a key factor affecting the reaction times in action anticipation tasks, and critical kinematic information, especially early arm swing movements, can be efficiently used only by experts. In contrast, amateurs displayed the highest accuracy and shortest reaction time under the high-information condition, with a significant drop in performance under medium- and low-information conditions, indicating that amateurs rely more heavily on complete visual information to make accurate predictions. These findings align with existing research, suggesting that sensitivity to subtle kinematics is the key to distinguishing skill proficiency among athletes [11]. The results of our study suggest that the anticipatory superiority of experts is not merely due to earlier information, but rather their ability to process earlier critical information, such as the initiation of opponents’ arm-swing movements. Their advanced skills in interpreting partial movement information enable experts to respond more quickly and accurately [29,30,31,32]. These results suggest that medium information is a critical factor for the accuracy of action anticipation tasks, and it represents a perceptual prediction advantage among expert athletes.

The analysis of alpha rhythms revealed distinct patterns between experts and amateurs under different information conditions, reflecting differences in attentional engagement and neural efficiency based on expertise. Experts exhibited the strongest alpha desynchronization under the low-information condition, indicating increased attentional engagement and cognitive workload when less information is available [26,33,34]. In contrast, amateurs showed the weakest alpha desynchronization under the medium-information condition. The differences in alpha desynchronization highlight the role of neural efficiency in high-level athletic performance. Studies have shown that alpha desynchronization is associated with enhanced attentional processes and neural efficiency during task performance [23,35]. Therefore, the greater alpha desynchronization observed in experts, particularly under challenging low-information conditions, suggests that they can maintain high levels of attention and cognitive control, even when critical action cues are limited. This neural efficiency allows experts to process and integrate information more effectively, supporting their superior performance in action anticipation tasks. These findings are consistent with previous research, indicating that expert athletes exhibit more pronounced alpha desynchronization in response to task-relevant stimuli [23,24,36]. The observed patterns of alpha activity in this study further validate the notion that expertise is associated with more efficient neural mechanisms for processing kinematic information during sports performance. These insights underscore the importance of attentional control and neural efficiency in distinguishing experts from amateur athletes.

The analysis of mu rhythms revealed significant differences between experts and amateurs, highlighting the mirroring mechanisms underlying the action anticipation abilities of expert volleyball players. Both groups exhibited the greatest mu rhythm desynchronization under the low-information condition, reflecting the increased demand of mirroring processing when minimal kinematic information is available. However, experts displayed greater mu rhythm desynchronization in the T3 and T4 time windows compared to amateurs. These time windows likely correspond to critical phases of spiking movements, where key kinematic information is present. The enhanced desynchronization observed in experts suggests that they are more effective at processing and integrating this crucial action information, reflecting stronger mirroring activity [19,28]. These patterns are consistent with previous studies, which have shown greater mu rhythm desynchronization in expert athletes while observing domain-specific actions [20,28,37]. Expert athletes possess refined neural mechanisms for action anticipation, an ability supported by the mirror neuron system [6,38,39,40,41,42]. Additionally, a notable finding was the negative correlation between mu rhythm power and anticipation accuracy. This relationship indicates that greater mu desynchronization is associated with better performance, highlighting the roles of the mirror mechanism and neural efficiency in expert anticipatory skills. Experts, who showed lower mu power during these critical windows, consistently achieved higher accuracy, suggesting that their superior performance is linked to more efficient neural processing. These findings reinforce the idea that expertise in sports involves enhanced mirror neuron activity and sensorimotor integration, enabling more accurate and rapid predictions.

Furthermore, studies suggest that anticipatory ability should be considered an important part of an athlete’s skill development [43]. The action anticipation performance of volleyball players develops with age and can be affected by mental fatigue [44,45]. Therefore, future research should focus on enhancing athletes’ abilities to process and utilize medium levels of kinematic information, as this has been shown to be particularly effective for enhancing expert performance. Additionally, incorporating neurofeedback techniques that target alpha and mu rhythms might further improve attentional engagement and kinematic processing, thereby enhancing athletes’ predictive capabilities [46,47,48]. 

## 5. Limitations 

Although the current study allowed us to identify key differences between volleyball experts and amateurs, it remains unknown whether these findings can be generalized to other sports. Furthermore, it is also not clear whether and how this critical ability can be developed through science-based training.

## 6. Conclusions

This study provides new insights into the cognitive and neural mechanisms underlying action anticipation in volleyball players, especially in the differences between experts and amateurs. Experts demonstrated shorter reaction times and higher accuracy, particularly under the medium-information condition, and exhibited distinct neural patterns in alpha and mu rhythms compared to amateurs. These results suggest that the abilities to process critical kinematic information and to maintain efficient attentional engagement are key factors contributing to the anticipatory superiority of volleyball experts. 

## Figures and Tables

**Table 1:** Demographic information.

	Expert (*n* = 26)	Amateur (*n* = 23)
Age	20.38 ± 1.82	21.26 ± 2.33
Years of Professional Training	7.80 ± 2.70	3.36 ± 1.84
Grades of Athletes	International Masters, *n* = 4 Grade 1 Athletes, *n* = 22	Grade 2 Athletes, *n* = 3 No grade, *n* = 20

**Table 2:** Accuracy and reaction times under different information conditions (average ± standard deviation).

	Information Condition	Expert	Amateur
		M ± SD	M ± SD
Accuracy	High	68.52 ± 10.93	65 ± 8.59
	Medium	68 ± 7.77	55.1 ± 9.14
	Low	61.91 ± 5.87	59.55 ± 8.71
Response Time	High	507.12 ± 150.91	528.83 ± 140.14
	Medium	475.09 ± 115.67	725.81 ± 262.68
	Low	596.42 ± 225.63	714.94 ± 324.93

## Data Availability

The original contributions presented in this study are included in the article; further inquiries can be directed to the corresponding author.
